# An Indoor Positioning Approach Based on Fusion of Cameras and Infrared Sensors

**DOI:** 10.3390/s19112519

**Published:** 2019-06-01

**Authors:** Ernesto Martín-Gorostiza, Miguel A. García-Garrido, Daniel Pizarro, David Salido-Monzú, Patricia Torres

**Affiliations:** 1 Electronics Department, Polytechnic School, University of Alcalá, 28871 Madrid, Spain; miguelangel.garcia@uah.es (M.A.G.-G.); daniel.pizarro@uah.es (D.P.); patricia.torress@edu.uah.es (P.T.); 2 Institute of Geodesy and Photogrammetry, 8093 Zurich ETH Zürich, Switzerland; david.salido@geod.baug.ethz.ch

**Keywords:** infrared sensors, cameras, indoor positioning, sensor fusion

## Abstract

A method for infrared and cameras sensor fusion, applied to indoor positioning in intelligent spaces, is proposed in this work. The fused position is obtained with a maximum likelihood estimator from infrared and camera independent observations. Specific models are proposed for variance propagation from infrared and camera observations (phase shifts and image respectively) to their respective position estimates and to the final fused estimation. Model simulations are compared with real measurements in a setup designed to validate the system. The difference between theoretical prediction and real measurements is between 0.4 cm (fusion) and 2.5 cm (camera), within a 95% confidence margin. The positioning precision is in the cm level (sub-cm level can be achieved at most tested positions) in a 4×3 m locating cell with 5 infrared detectors on the ceiling and one single camera, at distances from target up to 5 m and 7 m respectively. Due to the low cost system design and the results observed, the system is expected to be feasible and scalable to large real spaces.

## 1. Introduction

The framework of this proposal is positioning in indoor Intelligent Spaces. These kinds of Local Positioning Spaces (LPSs) are complex environments in which several sensors collect information, multiple agents share resources and position and navigation of mobile units appear as main tasks [1,2]. Under such complex conditions, having sensors with complementary capacities is necessary to fulfill all requirements satisfactorily.

At the end of this section we include a table (Table 1) with a comprehensive summary of the indoor positioning outlook presented in this introduction, according to accuracy and cost features, together with a description of the applications and some comments on their main strengths and weaknesses.

Depending on the application goals, a rough, but useful, classification may divide the indoor positioning systems in non-precise systems (from some tens of cm to 1 m level) or precise ones (1 to 10 cm). The former are typically human-centered applications in which m-level or room-level accuracy may be enough to fulfill the requirements (for example, localization of people or objects in office buildings). They are usually user-oriented applications based on portable technologies as mobile phones [3], Inertial Measurement Units (IMUs), etc. [4], or indoor local networks (WiFi, Zigbee, Bluetooth) [5]. They exploit the benefits of having an available infrastructure in the environment reducing, consequently, costs and sensor design effort at the expense of reaching lower precision than an ad-hoc sensorial positioning system, as the networks are originally conceived for communication purposes. They usually operate using Received Signal Strength (RSS) signals [6] to directly obtain distance estimations upon signal level measurements, or fingerprinting-based approaches relying on previously acquired radiomaps [5].

On the other side, in industrial environments, positioning systems for autonomous agents must be precise and robust. A cm-level positioning accuracy is needed when, for instance, an Automated Guided Vehicle (AGV) must perform different tasks such as carrying heavy loads inside a manufacturing plant [7,8]. In these spaces, the localization system (sensors and processing units) is very often deployed in the environment and needs careful specific design. Precise positioning of AGVs (and autonomous agents in general) is more visible today in industrial environments (for example, a manufacturing plant) than in civil ones (airports, hospitals, large malls, etc.) because they are more controlled spaces. In the second ones, due to human safety reasons together with the fact of being much more unpredictable spaces, their introduction is much slower. Unlike outdoor localization where Global Navigation Satellite Systems (GNSS) have imposed themselves, there is no dominant technology. Ultrasound (US) [8], cameras [9] and radio frequency (RF) [10,11], which also comprises Ultra Wide Band (UWB) technologies [12,13], and infrared (IR) have been mainly used so far. Alternatives with IR have the disadvantage of being directional, but they are very interesting when high precision is required in a channel without interference. All of them have strengths and weaknesses depending on the environmental conditions, the type of application and the performance required. They face several problems common to any LPS (occlusions, multipath, multiple users, etc.). Regarding the measuring principle, they mostly work with Time Of Flight (TOF) [12,14] measurements, which can in turn be Time Of Arrival (TOA) and Time Difference of Arrival (TDOA), Angle Of Arrival (AOA) [11], or RSS [15], as addressed in the next paragraphs and Table 1.

In this context, the interest of this proposal focuses in precise localization systems with two types of sensors for positioning a mobile agent: IR sensors and cameras. IR sensors are, as mentioned, an interesting option if precise localization and interference free channel is needed, providing secure communication capabilities. On the other hand, cameras are widely used in many applications (such as detection, identification, etc.), including indoor positioning applications [16]. In the type of environments mentioned above, low cost systems may be a need for scalability extension of the solution proposed to larger spaces. IR and camera solutions can meet this requirement, although IR need accurate ad-hoc design to deal with the very strong tradeoff between coverage (devices field of view), precision (Signal-to-noise ratio (SNR) achieved), real time response (integration time or filtering restriction) and cost [17]. The coexistence of IR sensors and cameras in a complex intelligent space can be very convenient. From the cooperative point of view cameras may carry out detection and identification of people, mobile robots or objects, environment modeling and also positioning. An IR system can perform localization and act as communication channel too [18]. Furthermore, if the sensors do not just cooperate but data fusion is carried out, the localization system improves with respect to two important aspects: first, the precision of the fused results is higher compared to the IR and camera ones, i.e., the variances of the position estimation obtained from fusion are lower than the variances of both sensors working independently [19]. Second, the fused position estimation presents high robustness because in case any of the sensors delivers low quality measurements (high dispersion due to bad measurement conditions or sensor failure), the fused variance keeps below both sensor variances, as close to the lowest one as the other. This second aspect is a key advantage for feasible and robust positioning during navigation.

Camera-based indoor localization systems work either with natural landmarks or artificial ones, the former being more widely used recently. This kind of approach requires ad-hoc offline processes to collect information about the environment and be stored in large databases [3,20,21,22]. The artificial landmark approach implies a more invasive strategy but, on the other hand, does not require a priori environmental knowledge [23,24]. With respect to the camera location, it is usually placed onboard the mobile agent [3,20,21,22,23], being a less common practice deploying the cameras in the environment infrastructure (which match better the conception of an intelligent space) [16,25]. Most works report a sub-meter precision like in [20,21], or in [3], where an online homography (same strategy as the one in our proposal) is used. Higher precisions are also achievable, as in [22], where a margin between 5 cm and 13 cm is reached with natural landmarks. A comprehensive review where different approaches and their features and performance can be found in [26]. In many works different sensors are used in a cooperative (not fusion) way, as in [20] with a camera and a Laser Imaging Detection and Ranging (LIDAR) or in [27] with camera and odometry, both for robot navigation applications, or [24] where ArUco markers [28] (widely known encoded markers, also used in this work) and an IMU cooperate for a drone navigation and landing application. There is no dominant approach in fusion of cameras with other sensors in positioning and navigation applications. In [6], a human localization system is proposed based on fusion of RF and IR pyroelectric sensors, with signal strength (RSS) measurements, is proposed. In [29], a fusion approach with inertial measurement units (IMUs) and Kalman filter for navigation purposes is shown. In [30] a fusion application with a similar approach as the one we present in this paper, combining the observations with covariance-matrix weights and running Monte Carlo simulations to test models and further comparison with real measurements is shown. An Interesting approach [31] where motion sensors and Bluetooth Low Energy (BLE) beacon are fused by means of a weighted sum.

Focusing on IR solutions, there are no dominant approaches in indoor localization systems either. They can be addressed in two ways: with collimated sources (mainly laser) performing a spatial sweep [32], or with static devices with open emission and opening angles as high as possible both in emission and reception [17,33]. In the first case, a high SNR is collected at the detectors but there is also greater optomechanical complexity requiring a precise scanning system [34,35], a structured environment (IR reflectors in known positions, with very precise alignment) and notably higher cost, in addition to demanding greater maintenance effort. With the second approach, which is the one used in the IR subsystem presented here, receivers collect lower SNR (hence engaging precision) but the system covers wider angle at lower cost. It implies a big design challenge to deal with this severe tradeoff. All these features (coverage, number of receivers needed, accuracy and cost) are key aspects towards scalability of a locating system to large spaces.

In the emerging context of Visible Light Communication (VLC) which tackles localization and communications making use of the same optical channel, the positioning systems exploit the IR device infrastructure as seen in [33] with TDOA measurements, or in [15] where the authors report precisions in the sub cm level with RSS, although both works provide only simulation results. Ref. [11] is another power-based approach plus AOA detection with three photodiodes, achieving precisions of 2 cm in a realistic setup. The precisions achieved in these works are valid in a measuring range up to approximately 2 m. Regarding IR and other sensors working together, many solutions are approached from a point of view of sensor cooperation or joint operation rather than, strictly considered, sensor fusion. In [36] passive IR reflectors are deployed in the ceiling while a camera boarded on a robot analyzes the scene under on/off IR controlled illumination, so that the joint performance is based on the comparison between both states’ joint response. A precision between 1 and 5 cm is achieved in a robot navigation application. In [37] a collaborative approach using a camera and an IR distance scanner is used for joint estimation of the robot pose, where the camera provides accurate orientation information from visual features while the IR sensor enhances the speed of the overall solution. While [36,37] are the most similar approaches to our proposal, given the use of IR and camera-based localization in a robotics context, both rely on significantly different approaches (detection of actively illuminated landmarks and SLAM, Simultaneous Localization and Mapping) and architecture (self-localization systems on-board of a mobile unit). Their main challenges and achieved performance are therefore hard to compare to the proposal described in this paper, as will be seen next. Another VLC application for positioning with three Light-Emitting Diodes (LEDs) and a fast camera, with fast code (from LEDs) processing, achieving precisions better than 10 cm in a 40 m${}^{2}$ area, can be found in [38]. A similar application to the latter, although making use of a mobile phone camera, is proposed in [39], showing decimeter level precisions. Many positioning systems are based on RSS measurements [15], or AOA [11,34], but precise optical telemetry is usually based on phase measurements [40].

Summarizing such a complex scenario with so high heterogeneity in the solutions proposed, we can ascertain that cm-level precision (below 10 cm) is quite difficult to achieve, not only in prepared experimental setups typically referred in the literature but, and specially, under realistic conditions in real environments. Home applications can cope with m-level and room-level accuracies while industrial ones may need (robust) performance down to 1 cm and need accurate ad-hoc solutions. Additionally, low cost solutions are very interesting in industry as, in many cases, large spaces must be covered (needing a high number of resources and devices for this purpose). The system we present here is intended to aim at meeting these requirements.

In this context, our proposal is a localization system developed with a phase-shift IR localization subsystem, composed of five receivers acting as anchors and an emitter acting as target, fused with a camera localization one, with a maximum likelihood (ML) approach. IR and camera models are developed and used for variance propagation to the final position estimation. Additive White Gaussian Noise (AWGN) hypothesis is supposed for the IR and camera observations in these models. For this, IR measurements must be mostly multipath (MP) free, which can be reasonably assumed in sufficiently large scenarios with low reflectivity of walls and ceiling, or by implementing MP mitigation techniques [41] or oriented sensors (as in [42] in an IR communications framework). The IR positioning system, with cm level precision, was successfully developed and shown in the past, and a model that relates the variances of position estimate to the target position was derived. The novelty lies in the development of another model to deduce observation variances and further propagation to camera position estimate by means of an homography, so that it can be used in the fused final position estimate. The novelty also lies in the fused sensor system itself as a measuring unit, which performs robustly delivering precisions in the cm level, and at the same time matches the models stated. To our knowledge there are no precise positioning systems with data fusion of a phase-shift IR system, developed ad-hoc with wide angle simple devices (IR LED emitter and photodiodes) and a single low cost camera detecting passive landmarks, performing with cm-level in ranges of 5 to 7 m.

In Section 2 the method description is presented. The IR sensor, camera and fusion estimation models are included in Section 3, Section 4 and Section 5 respectively, and evaluated with Monte Carlo simulations. Results on a real setup and the comparison with simulations to validate theoretical prediction from the models are presented in Section 6. A summary of key concepts derived from results discussion is included in the final conclusions in Section 7.

## 2. Method Description

**Figure 1:**
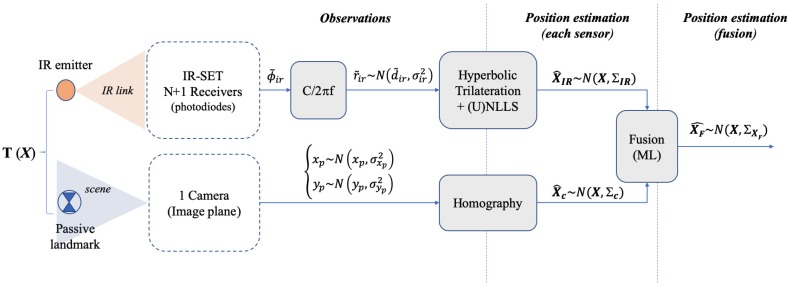
Block diagram description of the method.

A block level description of the strategy proposed is depicted in Figure 1. We consider a basic locating cell (BLC) where a target (a mobile robot) is to be positioned. This BLC is covered by an infrared positioning set (IR set hereafter) composed of several IR detectors and one single camera sharing the locating area. In this arrangement, the position X of the target T, defined as $X={\left(x,\;y,\;z_{T}\right)}^{T}$, is to be obtained. This target is an IR emitter for the IR sensor and a passive landmark for the camera. It lies in a plane with fixed (and known) height $z_{T}$, hence positioning is a 2D problem where the coordinates x,y of T in such plane are sought. After the IR and camera processing blocks, two position estimates, ${\hat{X}}_{\mathrm{IR}}$ and ${\hat{X}}_{c}$, are obtained from the IR and camera sensors respectively. ${\hat{X}}_{\mathrm{IR}}$ is attained by hyperbolic trilateration from differences of distances [45] and ${\hat{X}}_{c}$ is obtained by projecting the camera image plane onto the scene plane by means of a homography transformation. We assume both estimates are affected by bi-dimensional zero-mean Gaussian uncertainties, represented by their respective covariance matrices $\sum_{\mathrm{IR}}$, $\sum_{c}$. A fusion stage with a maximum likelihood (ML) approach is carried out yielding a final estimate ${\hat{X}}_{F}$ with expected lower uncertainty values than the original IR and camera ones. The information about the final precision is contained in the resulting covariance matrix $\sum_{F}$, which is addressed in Section 5.

A deep explanation of the IR positioning system (developed in the past) can be read in [17,45]. Some relevant aspects are recalled herein though, for better understanding of the proposal. In Figure 1, the IR BLC is composed of N + 1 receivers acting as anchors ($A_{i}$), one of them as common reference ($A_{r}$), being $A_{i}$ and $A_{r}$ the coordinate vectors $A_{i}={\left(x_{i},\;y_{i},\;z_{0}\right)}^{T}$ and $A_{R}={\left(x_{r},\;y_{r},\;z_{0}\right)}^{T}$, with z-coordinate fixed and known, equal to $z_{0}$. The coordinates of T are achieved by hyperbolic trilateration (HT) from the differences of distance measurements $\tilde{r_{ir}}$, from T to each one of the anchors $A_{i}$ (i=1…N) and from T to $A_{r}$. These $\tilde{r_{ir}}$ are obtained from differential-phase of arrival (DPOA) measurements, $\tilde{\phi_{ir}}$, between $A_{i}$ and $A_{r}$ [17]. Nevertheless, the quantities $\tilde{r_{ir}}$ will be named as observations hereafter as only a constant factor is needed to convert $\tilde{\phi_{ir}}$ into $\tilde{r_{ir}}$. Every observation $\tilde{r_{ir}}$ is assumed to have additive white Gaussian noise (AWGN) with variance $\sigma_{ir}^{2}$. The *x*,*y* variances of the position IR estimate (which are terms of $\sum_{\mathrm{IR}}$) result from the propagation of $\sigma_{ir}^{2}$ through the HT algorithm. This process, addressed in the next section, involves a set of positioning equations solved by non-linear least squares (NLLS) plus a Newton-Gauss recursive algorithm [46]. Note that considering the error as unbiased AWGN means there are no remaining systematic or other biasing error contributions (cancelled after error correction and calibration, or considered negligible compared with random contributions). This includes multipath (MP) errors, which means working in an MP free environment (large open spaces with low wall reflectivity or else, MP cancellation capabilities [41,47] or working with orientable detectors [42]).

On the other hand, the camera captures the scene and the center of a landmark placed on T is detected yielding its coordinates in the BLC locating plane (in the scene) after image processing (landmark identification and center detection algorithm) and further projection from the camera image plane to the scene. As already mentioned, this is carried out by means of a homography so that a bijective correspondence between the image plane and the scene plane makes it possible to express the real world coordinates as a function of pixel coordinates in the image plane (and reciprocally). The homography is applied to the rectified image after camera calibration.

Once the estimates ${\hat{X}}_{\mathrm{IR}}$ and ${\hat{X}}_{c}$ are known, after an ML fusion procedure we obtain ${\hat{X}}_{F}$ as

(1) $${\hat{X}}_{F}=W_{1}\cdot {\hat{X}}_{IR}+W_{2}\cdot {\hat{X}}_{c}$$

where the weights $W_{1}$ and $W_{2}$ depend on the IR and camera covariance terms, which in turn also depend on the *x*,*y* coordinates of T. Detailed models for the ${\hat{X}}_{\mathrm{IR}}$ and ${\hat{X}}_{c}$ estimates and their respective covariance matrices $\sum_{\mathrm{IR}}$, $\sum_{c}$ have been derived. We aim at two goals with these models: on the one hand, the models are the key theoretical basis to obtain the final fused estimation, as the covariance matrices acting as weights are obtained from said models. On the other hand, they are a very useful tool for the designer of the positioning system as low level parameters can be easily tuned and allow for evaluating the effect on precision, from sensor observation level to final fused estimation of position. It is also convenient to describe here the set of errors used to quantify the uncertainty of the position estimate, either delivered by simulation or by real measurements, and either referred to IR, camera or fusion. Considering an ellipse of *N* position estimations, the error metrics used are:

1. **Errors in x,y axes:** for every position in the test grid, the uncertainty in the *x* and *y* axis is assessed by the standard deviation in *x* or *y* respectively. This information is contained in the covariance matrix. This applies to Monte Carlo simulations or real measurements.
2. **Maximum and minimum elliptical errors:** the eigenvalues of the covariance matrix are the squares of the lengths of the 1-sigma confidence ellipsoid axis, and can be easily computed by a singular-value decomposition (SVD) of this covariance matrix. Hence, given a generic covariance matrix $\Sigma_{\theta }$ of a set of observations in $R^{2}$ with coordinates x,y, after this SVD decomposition the two eigenvalues $\lambda_{1},\lambda_{2}$ are obtained in a matrix $\Sigma_{\theta }^{{}^{\prime }}$. This matrices have the form:
  (2) $$\Sigma_{\theta }=\left[\begin{array}{cc} \sigma_{x}^{2} & \sigma_{xy}\\ \sigma_{yx} & \sigma_{y}^{2} \end{array}\right];\;\;\Sigma_{\theta }^{{}^{\prime }}=\left[\begin{array}{cc} \lambda_{1} & 0\\ 0 & \lambda_{2} \end{array}\right]=\left[\begin{array}{cc} \sigma_{1}^{2} & 0\\ 0 & \sigma_{2}^{2} \end{array}\right]$$
  The deviations with respect to these axes provide more spatial information about the uncertainty in each position. We will refer to these deviations as elliptical deviations, or elliptical errors. The complete spatial information of the confidence ellipsoid would include also the rotation angle of its axis with respect to, for instance, *x* axis. This is also easily computed, if needed, through the aforementioned SVD decomposition. In our case, the axis length is enough to assess the dimensions of the uncertainty ellipsoid and its level of circularity.

The criterion for considering one of them depends on the specific uncertainty description requirements as will be addressed along the paper as needed. The results delivered by Monte Carlo simulations, running the models aforementioned, will be compared with real measurements in a real setup.

## 3. Infrared Estimation Model

We developed an IR sensor in the past with cm-level precision in MP-free environments or with MP mitigation capabilities [45]. Three receivers (anchors) at least must be seen by the IR emitter (target) so that trilateration is possible. More receivers are usually used to increase precision as real measurements are always spoiled by additive noise. A 3×3 m IR BLC composed of five receivers was proposed in [17] so that three, four or five can be used in different configurations as needed (one of them being the common reference). For scalability purposes, this 5-anchor BLC must be linked with other BLCs and high level strategies must be defined too. Nevertheless, this question fell out of the scope of that work and is not considered here either.

**Figure 2:**
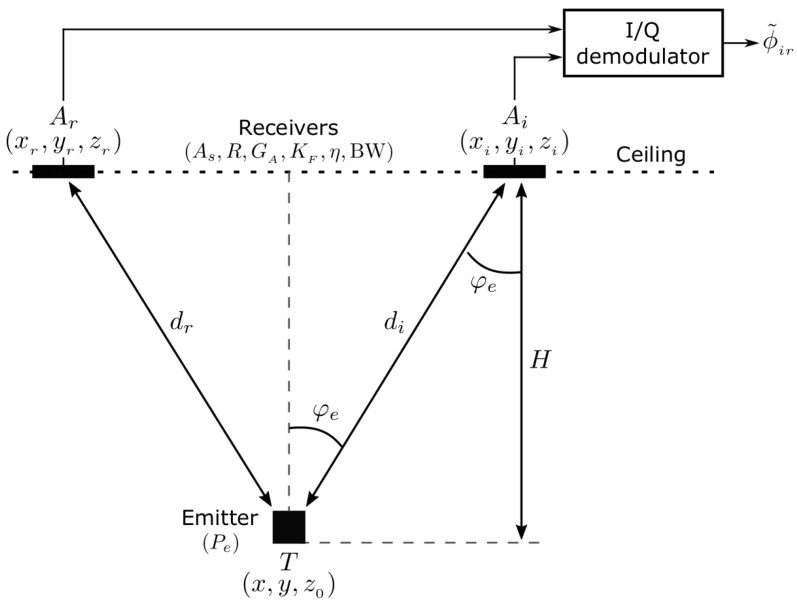
Infrared link representing a generic anchor $A_{i}$ (receiver) and the target T (emitter) in the IR-BLC. $A_{r}$ is the common reference in the basic locating cell (BLC).

In Figure 2 the IR link elements are depicted, summarizing the basic parameters involved in a DPOA measuring unit. Therefore, just the emitter placed at T and one of the receivers located at $A_{i}$ together with the reference $A_{r}$ are depicted, followed by an I/Q demodulator. As introduced in Section 2, and explained in detail in [17] the observation $\tilde{\phi_{ir}}$ is the ${\mathrm{DPOA}}_{ir}$ (phase difference between target T and receivers $A_{i}$ and $A_{r}$ respectively), directly obtained at the output of each I/Q demodulator. Regarding the IR model parameters (which encompasses radiometric, devices and electronics ones), the link T-$A_{r}$ is the same as any of the other T-$A_{i}$, as all receivers, including the reference one, are implemented with the same devices.

It is assumed that $\tilde{\phi_{ir}}∼N\left(\phi_{ir},\sigma_{\phi_{ir}}^{2}\right)$, where $\phi_{ir}$ is the DPOA true value. The corresponding observed differential range $\tilde{r_{ir}}$ is directly obtained as $\tilde{r_{ir}}=\frac{c}{2\pi f}\tilde{\phi_{ir}}$ and we also assume $\tilde{r_{ir}}∼N\left(d_{ir},\sigma_{ir}^{2}\right)$, where $d_{ir}$ is the distance-difference true value (i.e., $d_{ir}=\left|\left|T-A_{i}\right|\right|-\left|\left|T-A_{r}\right|\right|$, and $\left|\left|\cdot \right|\right|$ represents the 2-norm operator). In order to derive an IR model for the measurements, we consider $\tilde{r_{ir}}$ defined as $\tilde{r_{ir}}=\tilde{r_{i}}-\tilde{r_{r}}$, being $\tilde{r_{i}}∼N\left(d_{i},\sigma_{i}^{2}\right)$, $d_{i}$ the true value of the distance between T and $A_{i}$, and $\sigma_{i}^{2}$ the variance of $\tilde{r_{i}}$ (related to one single anchor $A_{i}$). This also applies to $\tilde{r_{r}}$ (with i=r). The variance term $\sigma_{i}^{2}$ can be modeled as a function of X and the IR measuring-system parameters, as will be shown further. This way, considering $\tilde{r_{i}}$ and $\tilde{r_{r}}$ as uncorrelated variables, we can compute $\sigma_{ir}$ as:

(3) $$\sigma_{ir}=\sqrt{\sigma_{i}^{2}+\sigma_{r}^{2}}$$

Note that while $\tilde{r_{ir}}$ are real observations from the measuring system (the I/Q demodulator directly delivers a phase-difference ${\mathrm{DPOA}}_{ir}$), $\tilde{r_{i}}$ and $\tilde{r_{r}}$ are virtual single-anchor observations, defined to derive the model.

As demonstrated in [48], the variance $\sigma_{i}^{2}$ can be expressed as the inverse of the signal to noise ratio at every anchor (${\mathrm{SNR}}_{i}$), and can be modelled as follows

(4) $$\sigma_{i}^{2}=\frac{\gamma }{{\mathrm{SNR}}_{i}}=K_{{}_{\mathrm{IR}}}\cdot d_{i}^{4}$$

where the factor γ is proved to be γ=1 [48], $d_{i}$ is the Euclidean distance (true value) between $A_{i}$ and T, and $K_{{}_{\mathrm{IR}}}$ is a constant encompassing all parameters of the IR system (including devices, electronics, noise and geometry), as follows:

(5) $$K_{{}_{\mathrm{IR}}}={\left(\frac{P_{e}\cdot A_{s}\cdot R\cdot G_{A}\cdot K_{F}\cdot K_{{}_{I/Q}}\cdot H^{2}}{\eta \cdot BW_{N}}\right)}^{-1}$$

All parameters appearing in (5) are known: $P_{e}$ is the IR emitted power per solid angle unit, $A_{s}$ and *R* are the photodiode sensitive area and responsivity respectively, $G_{A}$ is the i-v converter gain, $K_{F}$ is the filter gain (after i-v stage), $K_{{}_{I/Q}}$ is the I/Q demodulator gain (ideally unity gain) and *H* is the receiver’s height (measured from the emitter z-coordinate). The terms in the denominator, η and $BW_{N}$, are the noise power spectral density and noise bandwidth respectively. Consequently, the quantity in the denominator is the total noise power which, once the receiver parameters are fixed, is constant [17]. The distance $d_{i}$ in (4) is

(6) $$d_{i}=\sqrt{{\left(x-x_{i}\right)}^{2}+{\left(y-y_{i}\right)}^{2}+{\left(z_{0}-z_{T}\right)}^{2}}$$

also valid for $d_{r}$ making i=r. The relation in (4) is a very useful tool as it allows for expressing the variance terms as a function of the coordinates of distance $d_{i}$, hence as a function of the coordinates x,y of the sought target T (given that the coordinates of $A_{i}$ are constant) and fixed parameters grouped together in a single constant. Therefore, (4) and (5) establish the link between the random contributions in the observations and the coordinates X of the target. The covariance matrix of the observations $\tilde{r_{ir}}$ is

(7) $$\Sigma_{{}_{\mathrm{IR}}}=\left[\begin{array}{cccc} \sigma_{11}^{2} & \sigma_{12}^{2} & \cdots & \sigma_{1N}^{2}\\ \sigma_{21}^{2} & \sigma_{22}^{2} & & \\ \vdots & & \ddots & \\ \sigma_{N1}^{2} & & \cdots & \sigma_{NN}^{2} \end{array}\right]=\left[\begin{array}{cccc} \sigma_{1}^{2}+\sigma_{r}^{2} & \sigma_{r}^{2} & \cdots & \sigma_{r}^{2}\\ \sigma_{r}^{2} & \sigma_{2}^{2}+\sigma_{r}^{2} & & \\ \vdots & & \ddots & \\ \sigma_{r}^{2} & & \cdots & \sigma_{N}^{2}+\sigma_{r}^{2} \end{array}\right]$$

where every term $\sigma_{ii}^{2}$ in the diagonal represents the variance $\sigma_{ir}^{2}$ term defined in (3) of the observation $\tilde{r_{ir}}$. The covariance matrix is not diagonal, as the $d_{r}$ distance term is present in all the $\tilde{r_{ir}}$ terms. As said, the covariance matrix modelled this way links the position of the target in the navigation space with the uncertainty in the observations. Every diagonal term is computed as in (3) using the relation in (4), where $d_{i}$ and $d_{r}$ directly depend on the target coordinates through (6). The non-diagonal terms are directly deduced as a function of $d_{r}$ from expressions (4) to (6) with i=r.

The position X of the target is achieved by hyperbolic trilateration. From *N* observations $\tilde{r_{ir}}$ we can write

(8) $$\tilde{r_{ir}}=\left(d_{i}-d_{r}\right)+n_{ir}\;\;\;\;\left(i=1,\ldots N\right)$$

where $n_{ir}$ is an AWGN contribution with variance $\sigma_{ir}^{2}$. This is:

(9) $$\sqrt{{\left(x-x_{i}\right)}^{2}+{\left(y-y_{i}\right)}^{2}+{\left(z_{0}-z_{T}\right)}^{2}}-\sqrt{{\left(x-x_{r}\right)}^{2}+{\left(y-y_{r}\right)}^{2}+{\left(z_{0}-z_{T}\right)}^{2}}=\tilde{r_{ir}}-n_{ir}\;\left(i=1,\ldots N\right)$$

An estimate for $X={\left(x,y\right)}^{T}$ can be typically obtained by nonlinear (unweighted) least squares (NLLS) as follows:

(10) $${\hat{X}}_{\mathrm{IR}}=\underset{X}{\arg}\left(min\left(\sum_{i=1}^{N}\varepsilon_{i}^{2}\right)\right)=\underset{X}{\arg}\left(min\left(\varepsilon^{T}\varepsilon \right)\right)$$

where ε is an N × 1 column vector formed by the N residuals $\varepsilon_{i}$ (noise terms in (9)), i.e.,:

(11) $$\varepsilon_{i}=\left(\sqrt{{\left(x-x_{i}\right)}^{2}+{\left(y-y_{i}\right)}^{2}+{\left(z_{T}-z_{0}\right)}^{2}}-\sqrt{{\left(x-x_{r}\right)}^{2}+{\left(y-y_{r}\right)}^{2}+{\left(z_{T}-z_{0}\right)}^{2}}\right)-\tilde{r_{ir}}$$

which are the Gaussian uncertainty terms in the measurement of $\tilde{r_{ir}}$. (10) can also be typically solved iteratively with a Newton-Gauss algorithm, yielding a recursive solution:

(12) $${\hat{X}}_{{\mathrm{IR}}_{k+1}}={\hat{X}}_{{\mathrm{IR}}_{k}}+{\left(J_{\varepsilon }^{T}J_{\varepsilon }^{}\right)}^{-1}J_{\varepsilon }^{T}\varepsilon$$

The N × 2 matrix $J_{\varepsilon }^{}$ in (12) is the Jacobian of ε with respect to variables *x* and *y* (*z* is constant, as mentioned), with terms

(13) $$J\varepsilon_{{}_{i1}}=\frac{x-x_{i}}{d_{i}}-\frac{x-x_{r}}{d_{r}};\;\;J_{\varepsilon_{{}_{i2}}}=\frac{y-y_{i}}{d_{i}}-\frac{y-y_{r}}{d_{r}}$$

where $d_{i}$ and $d_{r}$ are computed as in (6). The covariance matrix of the NLLS estimator in (12) is then [49,50].

(14) $$\sum {}_{{\hat{X}}_{\mathrm{IR}}}={\left(J_{\varepsilon }^{T}\sum {}_{{\hat{X}}_{\mathrm{IR}}}^{-1}J_{\varepsilon }^{}\right)}^{-1}$$

computed at each x,y coordinates. The IR estimate obtained in (12) and the covariance matrix in (14) will be used in the final fusion estimate, in the form of (1). Note that the covariances in (7) are not obtained from experimental measurements but from the expressions derived from the IR model. Besides providing the weights in the fusion estimate, this covariance matrix can be also used for off-line simulations in order to evaluate the results under different configurations prior to real performance.

**Figure 3:**
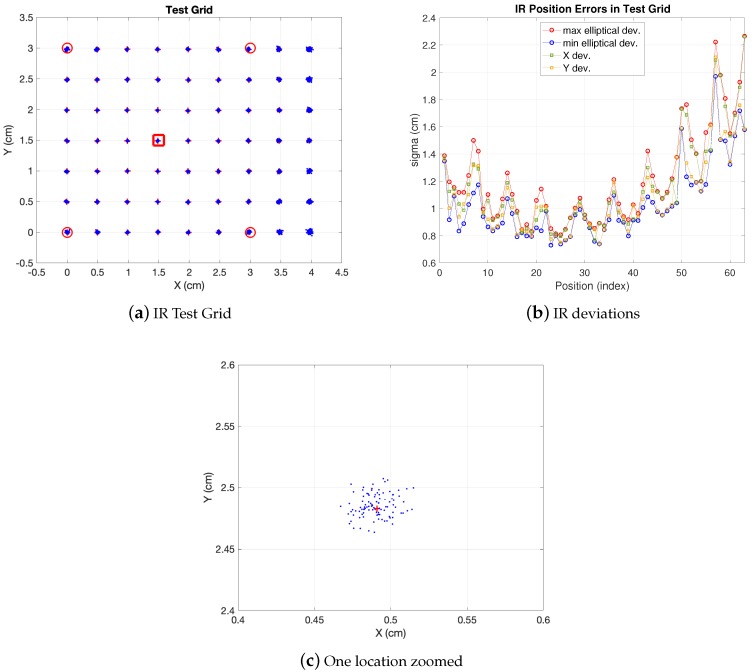
IR errors. Red markers indicate anchor projections on grid plane (red square is reference).

In Figure 3 the simulation results of the model explained for realistic conditions are displayed. As shown, in Figure 3a the whole considered test grid is depicted, with a 3×3 m IR set with 5 anchors placed at 2.7 m height (four in the corners and one in the center as common reference) covering a synthetic 4×3 m BLC. The additional area out of the IR set can be useful for transitions between BLCs or just for widening the area covered by one IR set, although with less precision (in the extra area out of the IR set the dispersion of the estimations increases). A set of 63 positions with 0.5 m separation between consecutive ones has been tested, with 100 realizations at each position. One of the locations is zoomed so that the observations cloud can be seen. The elliptical errors are evaluated at every position upon the observation ellipse (as the one zoomed). The IR link features in this simulation are: emitting power ($P_{e}$) 75 mW/sr, detector area ($A_{s}$) 100 mm${}^{2}$, responsivity (*R*) 0.64, i-v gain factor ($G_{A}$) $33\times 10^{3}$, filter gain ($K_{F}$) and I/Q demodulator gain ($K_{{}_{I/Q}}$) are both unity factor, noise power spectral density (η) $1.34\times 10^{-11}$ W/Hz and noise equivalent bandwidth ($BW_{N}$) 30π/2 Hz. These are the values for the parameters appearing in (5) with the geometrical parameter *H* set to 2.7 m. In Figure 3b the values of these errors along the whole test cell are displayed (indexed starting at the left-bottom corner of the grid and growing in columns up to the upper right corner). As shown, under the conditions aforementioned, less dispersion is observed in the central area. The uncertainty in x,y axis are in a margin between 1 cm and 2.3 cm and between 0.9 cm and 2.1 cm values for $\sigma_{x}$ and $\sigma_{y}$ respectively. The 68% confidence ellipse is defined by axis with σ values between 1.15 cm and 2.2 cm and between 0.7 cm and 2 cm (a 95% confidence ellipse would be defined by two times these σ values). The closeness of the deviations in the ellipse axis compared to x,y is due to the central geometry derived from choosing the reference anchor in the center and using the five anchors in the BLC. More ellipticity would be observed in choosing another reference anchor or using less anchors in the BLC.

The IR set defines an area enclosed by the perimeter defined by the four vertices at the external anchors. This is useful for modular scaling of a larger positioning environment. Nevertheless, as can be seen in the figure, a larger area (BLC) can be covered by the same anchor deployment. This may be quite convenient when scaling the LPS as said, for locating the mobile robot when navigating between consecutive BLCs. It allows for different high level design strategies making it possible, for instance, to separate the anchors as much as possible if cost reduction is a need (at the cost of lower precision). This applies also for the camera coverage as will be seen. However, the design strategy of a larger space linking several BLCs lies out of the scope of this paper. Finally, any other configuration in which any of the parameters is changed (photodiode sensitive area, emitted power, etc) can be easily evaluated. The real tests reported in the results section are carried out with similar values as the ones synthetically generated in Figure 3.

## 4. Camera Estimation Model

The camera is placed in the BLC at fixed co-ordinates and not necessarily at the same height as the IR anchors. It may lay out of the polygon formed by the IR receivers and, although in this work both sensors are tested with the same (target) test-position grid, it might cover, if needed, a different target-positioning area than the IR sensors (the fusion would be, nevertheless, carried out in the intersection of both areas). Let us distinguish the four procedures that take place for proper camera performance in the LPS.

**Figure 4:**
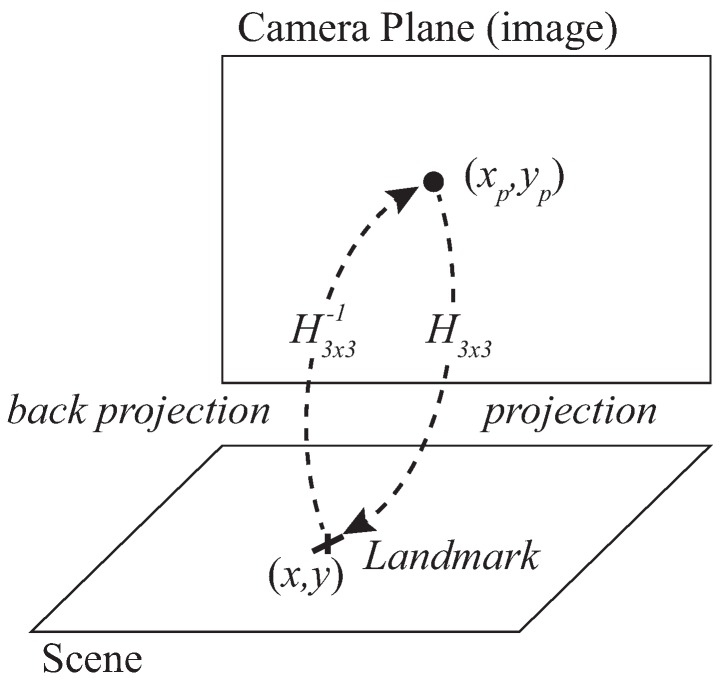
Camera sensor: homography relation between scene and image.

- **Calibration**: it is carried out, customarily, by means of a calibration pattern, in order to obtain the intrinsic and extrinsic camera parameters. Once these parameters are known, the coordinates of the detected target from the scene can be obtained in the image. Calibration has a big impact on errors in the final positioning. In any case, this is a standard stage in any camera-based landmark recognition application.
- **Homography parameters computation**: a homography is established between the image and scene planes (as already mentioned, the scene is also a plane of known height). It is a reciprocal transformation from image to scene. The coefficients of the matrix H for such transformation are obtained by means of specific encoded markers (named H-markers hereafter). This process (with low computational and time cost) allows for having a bijective relation between camera and navigation planes. This way, positioning can be easily carried out with one single camera. As explained in Section 2, the uncertainty in the image capture is propagated to uncertainty in the position in the scene through H. For setup characterization purposes, we have carried out this process off line but, in real performance, it can normally be implemented on line.
- **Target detection** (Projection): a landmark placed on the target is detected by image processing so that the position of the target is obtained in the image plane in pixel coordinates. For simplicity, we will refer hereafter to *projection* when considering the projection path from image to scene (with matrix H) and to *back projection* in the opposite sense (scene to image, with $H^{-1}$), as represented in Figure 4.
- **Back projection**: the back projection stage projects the position of T scene to the camera image by means of the inverse homography matrix $H^{-1}$ matrix. Back projection is used for simulation of the camera estimation model.

In Figure 4 the true position of the landmark is represented by the coordinate vector X (same as for IR sensor) and the captured position in the camera plane is $X_{p}^{t}\left(x_{p},y_{p}\right)$ where $x_{p}$ and $y_{p}$ are the x,y coordinates in such camera plane in pixelic units (index *p* stands for *pixelic* hereafter). A homography can be defined between the scene and camera planes, so that the relation between the coordinates of the landmark in the scene, $X^{t}\left(x,y\right)$, and $X_{p}^{t}\left(x_{p},y_{p}\right)$ in the image can be written as (15):

(15) $$\left(\begin{array}{c} sx\\ sy\\ s \end{array}\right)=H\left(\begin{array}{c} x_{p}\\ y_{p}\\ 1 \end{array}\right)=\left(\begin{array}{ccc} h_{11} & h_{12} & h_{13}\\ h_{21} & h_{22} & h_{23}\\ h_{31} & h_{32} & h_{33} \end{array}\right)\left(\begin{array}{c} x_{p}\\ y_{p}\\ 1 \end{array}\right)$$

In (15) H is the homography transformation matrix, the terms $h_{ij}$ of which are specific for every scene and camera planes (hence camera location) and must be obtained accordingly. The homography matrix is a 3×3 matrix but with 8 DoF (degrees of freedom) because it is generally normalized with $h_{33}=1$ or $h_{11}^{2}+h_{12}^{2}+h_{13}^{2}+h_{21}^{2}+h_{22}^{2}+h_{23}^{2}+h_{31}^{2}+h_{32}^{2}+h_{33}^{2}=1$ since the planar homography relates the transformation between two planes with a scale factor *s* [51]. This way, the coordinates in the scene are obtained as follows:

(16) $$\left\{\begin{array}{c} x=\frac{sx}{s}\\ y=\frac{sy}{s} \end{array}\right.\to \left\{\begin{array}{c} x=\frac{h_{11}x_{p}+h_{12}y_{p}+h_{13}}{h_{31}x_{p}+h_{32}y_{p}+h_{33}}\\ y=\frac{h_{21}x_{p}+h_{22}y_{p}+h_{23}}{h_{31}x_{p}+h_{32}y_{p}+h_{33}} \end{array}\right.$$

Let us define the 2-variables function $F_{H}$ defined by (16) so that $X=F_{H}\left(X_{p}\right)$. Given pixelic variances $\sigma_{x_{p}}^{2}$ and $\sigma_{y_{p}}^{2}$ and considering the camera observations with behavior $x_{p}∼N\left(x_{p},\sigma_{x_{p}}^{2}\right)$ and $y_{p}∼N\left(y_{p},\sigma_{y_{p}}^{2}\right)$, the jacobian of $F_{H}\left(J_{F_{H}}\right)$ for further variance propagation is:

(17) $$J_{F_{H}}=\left(\begin{array}{cc} \frac{\partial x}{\partial x_{p}} & \frac{\partial x}{\partial y_{p}}\\ \frac{\partial y}{\partial x_{p}} & \frac{\partial y}{\partial y_{p}} \end{array}\right)=\left(\begin{array}{cc} \frac{h_{11}\cdot D-h_{31}\cdot F_{1}}{D^{2}} & \frac{h_{12}\cdot D-h_{32}\cdot F_{1}}{D^{2}}\\ \frac{h_{21}\cdot D-h_{31}\cdot F_{2}}{D^{2}} & \frac{h_{22}\cdot D-h_{32}\cdot F_{2}}{D^{2}} \end{array}\right)$$

where the terms $F_{1}$, $F_{2}$ and *D* are computed as:

(18) $$\left\{\begin{array}{c} F_{1}=h_{11}x_{p}+h_{12}y_{p}+h_{13}\\ F_{2}=h_{21}x_{p}+h_{22}y_{p}+h_{23}\\ D=h_{31}x_{p}+h_{32}y_{p}+h_{33} \end{array}\right.$$

Given $\left(x_{p},y_{p}\right)$, pixelic coordinates of the captured landmark, yielding a corresponding position estimation ${\hat{X}}_{c}$, the covariance matrix of the camera estimation of position is:

(19) $$\Sigma_{{\hat{X}}_{p}}=J_{F_{H}}\cdot \Sigma_{p}\cdot J_{F_{H}}^{T}$$

**Figure 5:**
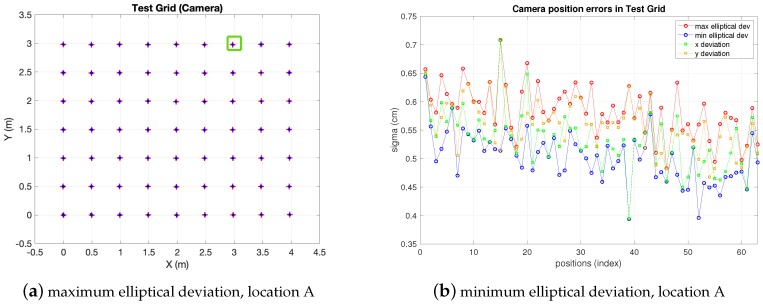
Camera errors (*x*,*y* and elliptical deviations), green square represents camera projection on grid plane.

Obtained from the covariance matrix of the camera observations $\Sigma_{p}$

(20) $$\Sigma_{p}=\left(\begin{array}{cc} \sigma_{x_{p}}^{2} & 0\\ 0 & \sigma_{y_{p}}^{2} \end{array}\right)$$

and the jacobian described in (17). It is noteworthy to remark here that in addition to the projection-homography (camera to scene) described in (15), it is also necessary to work with the back projection (scene to camera), defined by $X_{p}=F_{H^{-1}}\left(X\right)$, for evaluation of the method through simulation. To do so, synthetic true positions of the target in the grid are generated and back projected to the camera plane by means of $H^{-1}$. Next, in the camera image, synthetic realizations with a bidimensional Gaussian distribution are also generated, with center in the true positions back projected to the image plane before. This cloud of image points is then projected to the scene by means of H. This Monte Carlo simulation allows evaluation of errors in the scene given certain known values of pixelic errors and knowledge of H matrix. It must be noted that the H matrix depends on the camera location and, therefore, it must be obtained specifically for such a location. In Figure 5 the same test-grid as the IR one of previous section (a 4×3 m rectangular cell with 63 positions separated 0.5 m each other) is evaluated running a simulation as explained in the previous paragraph. The camera, represented by a green square in the figure, is set at an arbitrary position at 3 m high with respect to the horizontal plane. Operating with a single camera it may be interesting to cover a wide angle for inexpensive scalability to large areas, at the cost of more distortion (on the contrary, a camera placed in the center experiences less distortion, but less coverage too).

Supposing realistic uncertainty values $\sigma_{x_{p}}$ and $\sigma_{y_{p}}$ equal to 5 pixels (they could also be different from each other) and the following real H coefficients ($h_{11}=2.9\times 10^{-2}$; $h_{12}=-9.9\times 10^{-1}$; $h_{13}=1.1\times 10^{3}$; $h_{21}=9.9\times 10^{-1}$; $h_{22}=3.3\times 10^{-2}$; $h_{23}=-1.7\times 10^{-3}$; $h_{31}=1.2\times 10^{-5}$; $h_{32}=-1.7\times 10^{-5}$; $h_{33}=8.3\times 10^{-1}$), the precision of the position in the scene can be seen in Figure 5: the deviation in x,y axis are between 0.46 cm and 0.7 cm and between 0.47 cm and 0.65 cm values respectively, being the 68% confidence ellipse within margins of 0.5 cm and 0.66 cm and between 0.4 cm and 0.65 cm (ellipse axis respectively).

Note the decrease in the deviations as the test positions get closer to the camera. Other tests may be run in the same manner with the camera in any other location (yielding another matrix H).

## 5. Fusion of Camera and IR Sensors

The fusion estimation $\hat{X_{F}}$ is reached by a maximum likelihood (ML) approach [19], i.e., maximizing, with respect to X, the joint probability of having $\hat{X_{\mathrm{IR}}}$ and $\hat{X_{c}}$ estimates given a true position X. This is, given that both estimates are independent of each other:

(21) $$\hat{X_{F}}=\underset{X}{\arg}[\max\left(p\left(\hat{X_{\mathrm{IR}}},\hat{X_{c}}|X\right)\right]=\underset{X}{\arg}[\max\left(p\left(\hat{X_{\mathrm{IR}}}|X\right)\cdot p\left(\hat{X_{c}}|X\right)\right]$$

The IR and camera estimates are $\hat{X_{\mathrm{IR}}}∼N\left(X,\Sigma_{\mathrm{IR}}\right)$ and $\hat{X_{c}}∼N\left(X,\Sigma_{c}\right)$ respectively, where X is the true position of *T* and $\Sigma_{\mathrm{IR}}$, $\Sigma_{c}$ are the IR and camera covariance matrixes defined by (12) to (14) and (19) to (20), respectively. Consequently, $\hat{X_{\mathrm{IR}}}$ is described by:

(22) $$\hat{X_{\mathrm{IR}}}=\frac{1}{\sqrt{2\pi }{\left|\Sigma_{\mathrm{IR}}\right|}^{\frac{1}{2}}}e^{-\frac{1}{2}{\left(\hat{X_{\mathrm{IR}}}-X\right)}^{T}\Sigma_{\mathrm{IR}}^{-1}\left(\hat{X_{\mathrm{IR}}}-X\right)}$$

and $\hat{X_{c}}$:

(23) $$\hat{X_{c}}=\frac{1}{\sqrt{2\pi }{\left|\Sigma_{c}\right|}^{\frac{1}{2}}}e^{-\frac{1}{2}{\left(\hat{X_{c}}-X\right)}^{T}\Sigma_{c}^{-1}\left(\hat{X_{c}}-X\right)}$$

Introducing (22) and (23) in (21), it follows that $\hat{X_{F}}$ is found as:

(24) $$\hat{X_{F}}=\underset{X}{\arg}\left[\min\left({\left(\hat{X_{\mathrm{IR}}}-X\right)}^{T}\Sigma_{\mathrm{IR}}^{-1}\left(\hat{X_{\mathrm{IR}}}-X\right)+{\left(\hat{X_{c}}-X\right)}^{T}\Sigma_{c}^{-1}\left(\hat{X_{c}}-X\right)\right)\right]$$

Yielding the fusion estimate, computed as follows:

(25) $$\hat{X_{F}}=\left(\Sigma_{\mathrm{IR}}^{-1}+\Sigma_{c}^{-1}\right)\cdot \left(\Sigma_{\mathrm{IR}}^{-1}\hat{X_{\mathrm{IR}}}+\Sigma_{c}^{-1}\hat{X_{c}}\right)$$

**Figure 6:**
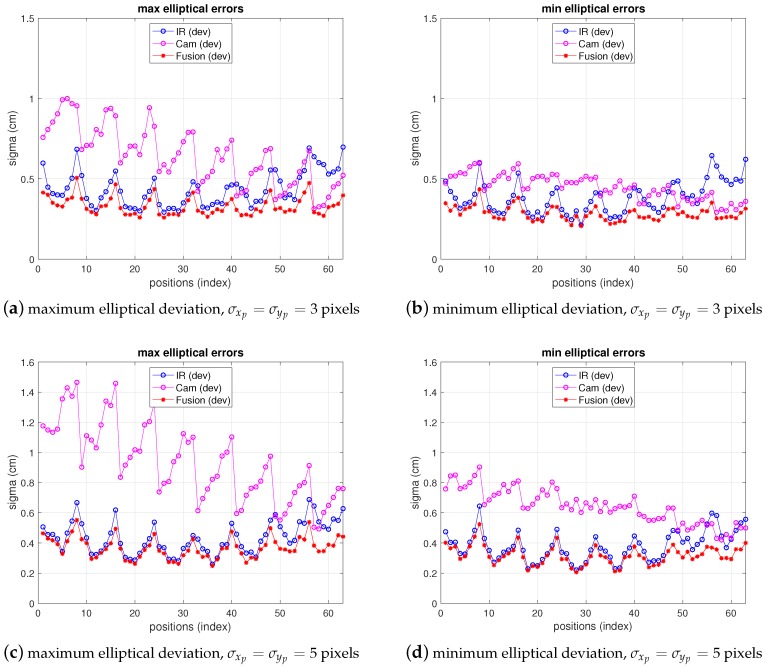
Fusion simulation errors.

Regarding covariance X-dependence, note the approach followed herein in reaching (25): the joint probability is maximized in (21) with respect to X, considering covariances as constant. Covariances depend on the position X as seen in the derivation of IR and camera models in previous sections. However, to simplify the optimization process, a good tradeoff to find the solution is solving the ML problem as stated in (21) to (25), where X is the optimization variable, and compute the IR and camera covariance matrixes in 25, defined as in (7) to (14) and (17) to (20) respectively, using the position estimation delivered either by the IR sensor or by the camera sensor. The criterion for choosing one of both can be defined as needed depending on the application. By default, if no other requirement is set, the one with minimum variance (estimated at X with each respective model) is chosen. In static conditions this would be a good solution; in dynamic (navigation) conditions, we can compute the fusion-position X[k] by using the covariance matrixes from X[k−1]. If the position update velocity is high enough this is also a good fast real-time solution. The results of fusion simulations are depicted in Figure 6. In this figure, the tests reproduce the conditions of the respective IR and camera simulations shown in the previous two sections. The standard deviations in the x,y axis, together with the maximum and minimum variances (68% confidence ellipse axis) are displayed for camera, IR and fusion results. As expected, the deviations delivered by the fusion estimate are lower than any of the two single sensors. The more one of the deviations (IR or camera) increases, the more fusion variance approaches the lowest one.

## 6. Results

In this section, a set of results obtained from real measurements conducted in a real basic locating (BLC) to assess the sensors performance and the models derived, is shown. First, IR and camera results are shown independently in Section 6.2 and Section 6.3 respectively. The fusion results are addressed next in Section 6.4. Two approaches are considered to determine the camera observation variances that appear in the fusion estimation. One of them is closer to the theoretical description, based on unbiased Gaussian uncertainty of the observations and the other one consists in defining a new standard deviation upon the real measurements (which are not purely unbiased). The latter differs more from the theoretical assumptions but approaches better the real behavior and allows for having a practical design tool with the same theoretical basics derived in previous sections.

The section starts with the description of the setup used for tests. Results from measurements are shown next and finally, a comparative analysis between measurement results and theoretical prediction is included.

In order to facilitate a faster knowledge of the results behavior we include some tables in the different sections as explained next. In Section 6.2 and Section 6.3 (IR and camera respectively): a summary of the precision (defined upon standard deviation) in the BLC, together with two indicators to have a better view of the behavior (shape) of the estimation clouds, are presented in a table. These indicators are defined in Section 6.2 (IR) and further used in Section 6.3 (camera) too. In Section 6.4 (results of fusion, also compared with IR and camera) the summarizing table is focused on the precision levels, representing the elliptical deviations explained in Section 2.

### 6.1. Setup

**Figure 7:**
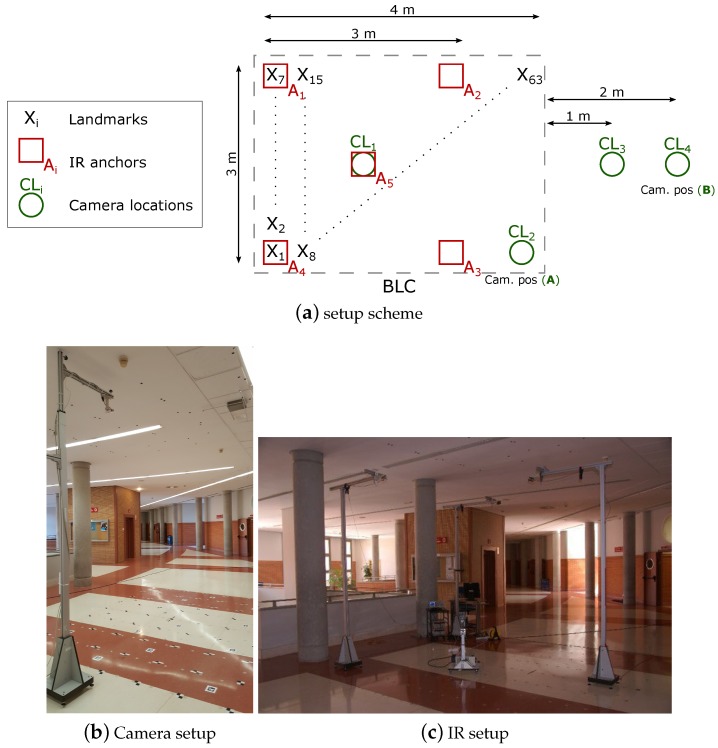
Setup for measurements.

The setup is depicted in Figure 7. A 4×3 m rectangular BLC is covered by a set of five IR anchors deployed in a 3×3 m square inside the BLC (four at the corners and one in the center) at 2.7 m height and one single camera, placed at 3 m height. The 3×3 m IR set covers the full 4×3 m BLC, so that the area out of the IR set can be considered as a transition zone between different BLCs in an eventual larger space. The test grid is composed of 63 (9×7) test positions separated 50 cm each other. At every position in the grid an IR emitter and a landmark are placed, acting as targets. An amount of 200 observations have been taken (phase shifts with IR and images with camera) yielding 200 IR and camera position estimations respectively and 200 fusion estimations. In addition, five illumination controlled levels where included in the tests, at every test-position and every camera location (therefore, in fact, the total number of camera images at every grid position is 200×4). Although the IR anchors could be flexibly chosen (keeping a minimum of three, necessary for trilateration), we work here with the full 5-IR anchor set with the reference in the center. The camera has been placed at four different locations in order to analyze the tradeoff between covered area (by the camera) and precision. We will show here the deviations obtained at these four positions and, next, detailed results are focused on the camera at two of these locations, *A* and *B*, (shown in Figure 7), which represent two extremes regarding such precision versus coverage tradeoff. The devices are selected to fulfill low cost requirements while fulfilling performance conditions. All features of the test bench are summarized in Table 2, including devices, BLC configuration and test conditions (notation and configuration indexes or labels corresponding to those in Figure 7). The IR system works with an IRED as emitter (and in turn positioning target) and a photodiode as receiver (in turn positioning anchors), as indicated in the table. All electronic circuits, including the stages for signal conditioning and phase measuring had already been specifically designed for this purpose in past projects. The camera is also an inexpensive one with the sensor shown in the table and a Raspberry Pi 3 Model B as processing unit. The landmarks used for target detection are also shown in Figure 7.

The IR measurements and positioning system performance had already been developed and shown in the past [45]. The camera data was collected for fusion purposes, which constitutes the core of the results presented in this paper.

### 6.2. Infrared Measurements

**Figure 8:**
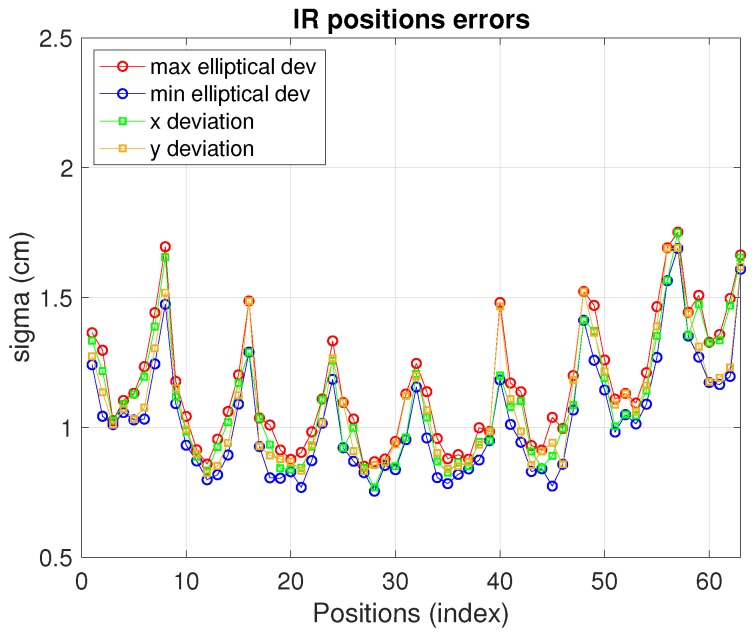
Infrared sensor positioning errors.

The IR real measurements, under the conditions described in Table 2, deliver the positioning errors displayed in Figure 8. In this case the configuration with higher precision has been chosen (five receivers with reference in the center). Other possible configurations might need less resources (lower number of anchors and/or more separated) and, hence, implying lower costs at the expense of less precision too. As can be seen, for this IR setup the elliptical deviations are between 0.75 cm and 1.75 cm. The maximum and minimum elliptical deviation values are close to each other (and also close to the *x* and *y* deviations) due to the symmetry and circularity of the geometry defined by the IR set chosen) as can be seen in Table 3.

An indicator to assess the shape of the estimation clouds and to have a better geometrical view of the results are introduced here: a *dissimilarity index (DI)* which will be useful to quantify, in percentage, the level of closeness of the different standard-deviation of the results shown in Figure 8. We first define the DI for two arbitrary matrices M and N of same dimensions:

(26) $$DI=\frac{{∥M-N∥}_{F}}{{∥M∥}_{F}}\cdot 100$$

which can vary from 0% (identical matrices) to *∞*. In (26) $||\;{\left|\right|}_{F}$ is the Frobenius norm, also applicable to one dimension vectors. We use (26) to compare, in pairs, the standard deviations in the original x,y axis, $\sigma_{x}$, $\sigma_{y}$, with the elliptical ones defined in Section 2. For this, a 63×1 vector containing the values of σ at each position is built (one vector for each of the four axis), being M in (26) either the $\sigma_{x}$ vector or the $\sigma_{y}$ one. Note that $||\;{\left|\right|}_{F}$ is equal to the classical Euclidean $||\;{\left|\right|}_{2}$ norm if applied to a vector. We nevertheless introduce the general definition for matrices as it would have a wider range of application, if needed, in other works (e.g., if all the covariance matrices are wished to be compared). Here, it is more convenient to see how close the axis deviations are from each other independently. In addition, note that a *DI* equal to 0% in both axis would mean that both deviation pairs are identical (i.e., the ellipse is oriented in the x,y original axis). In this case we would not be able to ascertain the circularity of the estimation cloud. Hence, as complementary information, we also define a *circularity index (CI)* as:

(27) $$CI=\frac{\lambda_{i_{min}}}{\lambda_{i_{max}}}\cdot 100$$

where $\lambda_{i_{min}}$ and $\lambda_{i_{max}}$ are, respectively, the minimum and maximum value of the eigenvalues pair $\left\{\lambda_{1},\lambda_{2}\right\}$ appearing in (2). This index is evaluated at every position in the BLC grid. In Table 3 the *CI* average in the CBL is included (note that *CI* itself could be enough to ascertain the ellipticity of the estimations but both *CI* and *DI* defined provide more detailed information about the estimations’ shape and behavior). In this table, the elliptical deviation 2σ values in the major and minor axis are also included. Namely the maximum (worse case) and average values in the whole CBL are shown. The 2σ values are chosen as they define the 95% confidence ellipsoid.

### 6.3. Camera Measurements

**Figure 9:**
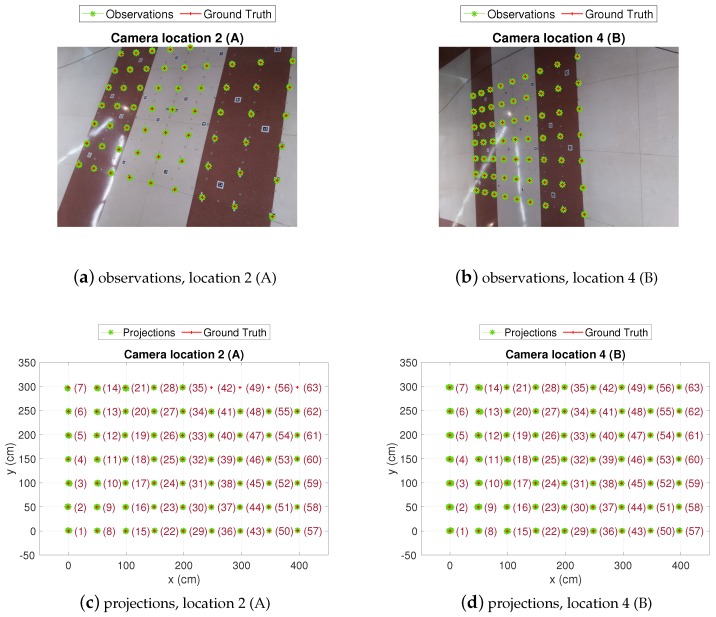
Test-grid and camera estimations view.

**Figure 10:**
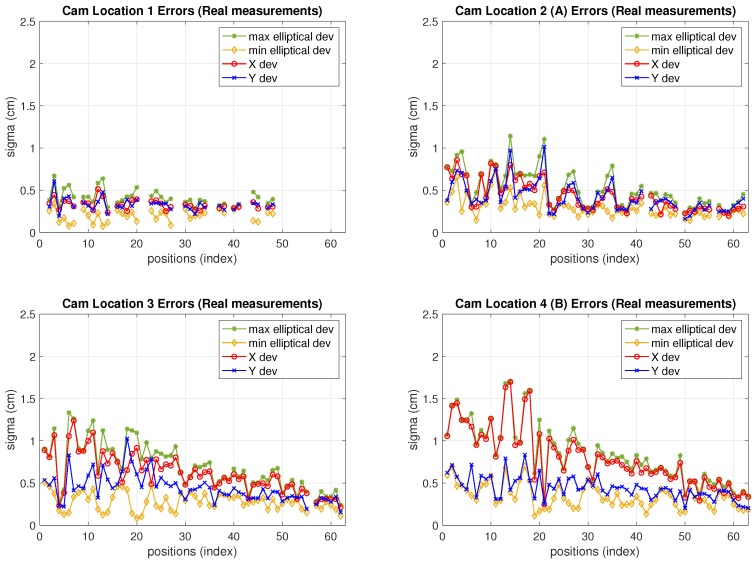
Camera sensor positioning errors (Real measurements).

In Figure 9 the position estimations in the CBL obtained from the camera observations at locations *A* and *B* are shown ($CL_{2}$ and $CL_{4}$ in Figure 7, respectively). The whole grid can be seen, with 200 estimations at every test position. As expected, in location *A*, the dispersion of the estimation clouds is lower because the camera is closer to the target. However, from location *A* the whole grid is not covered by the camera field of view and some test positions fall in blind areas. On the contrary, from location *B* the full BLC is covered by the camera, though dispersion is higher (less precision than in location *A*). In any case, as will be discussed in the next paragraph, and shown in Figure 10 and Table 4, at either of the camera locations *A* or *B*, the dispersion of the estimations increases as the distance from target to camera increases (this happens in the left-up direction from *A* and left direction from *B*).

In order to have a comprehensive view of the camera performance, in Figure 10 the standard deviations for all camera locations (not only *A* and *B*, but all locations 1 to 4) at every test position are depicted. The four standard deviation values defined in Section 2 are displayed (x,y deviations and elliptical ones). As can be seen, the elliptical deviations are in a range between 0.1 cm and 1.7 cm, increasing as the camera is more separate from the targets (progressively from locations 1 to 4) being similar at locations 1 and 2 as can be seen in Table 4. This would define 2σ (95%) confidence ellipsoids with axis between 0.2 cm and 3.4 cm. In addition, the x,y deviation values increasingly differ from each other, and from the elliptical ones too, from locations 1 to 4. Due to the more distortion as the camera gets further from the grid towards location 4, the estimation clouds get more elliptical. The discontinuities in the graphs at locations 1 and 2 correspond to blind grid positions not covered by the camera. In the same manner as explained in previous Section 6.2 (IR measurements) the Table 4 summarizes the information about estimation-clouds’ shape and measurements precision.

Next, in order to obtain specific information about pixelic deviations, which are needed to compute the fusion estimate according to the method derived in previous sections, the whole set of collected data is represented in Table 5. It contains the standard deviation values, in pixelic units, in the image x,y axis for each of the camera locations. In the table, the information is summarized showing the maximum and minimum values in the grid, as well as the average of such deviations (for all illuminations).

Focusing on locations *A* and *B*, the maximum pixelic deviations in the x,y image axes are: $\sigma_{x_{p}}=4.1$, $\sigma_{y_{p}}=4.5$, at *A*, and $\sigma_{x_{p}}=4.1$, $\sigma_{y_{p}}=3.9$ at *B*. It must be taken into account, in order not to misunderstand the table information, that a higher pixelic deviation does not necessarily mean a higher distance deviation in the scene (resolution worsens as camera-target distance increases). In fact, as seen in Figure 10, deviations in location 4 (labeled as *B*) are clearly higher than in location 2 (labeled as *A*), while the pixelic deviations are similar in both cases. In the next section we will introduce these values of pixelic deviations in the expressions in (19) to obtain the variance of $\hat{X_{c}}$ and compute the fusion estimate as in (24). The choice of deviation values is a key point, as discussed further. The terms of H needed in (19) to propagate pixelic variance with the function $F_{H}$, are obtained from an off-line characterization process to define an homography from point correspondences between the ArUco markers and image [28]. It must be remarked here that in Table 5 only the standard deviations are displayed, hence any systematic or any other biased error is not reflected there. This topic will be addressed in the fusion results section as, actually, a higher error is obtained from camera results due to such type of errors.

### 6.4. Fusion Results

**Figure 11:**
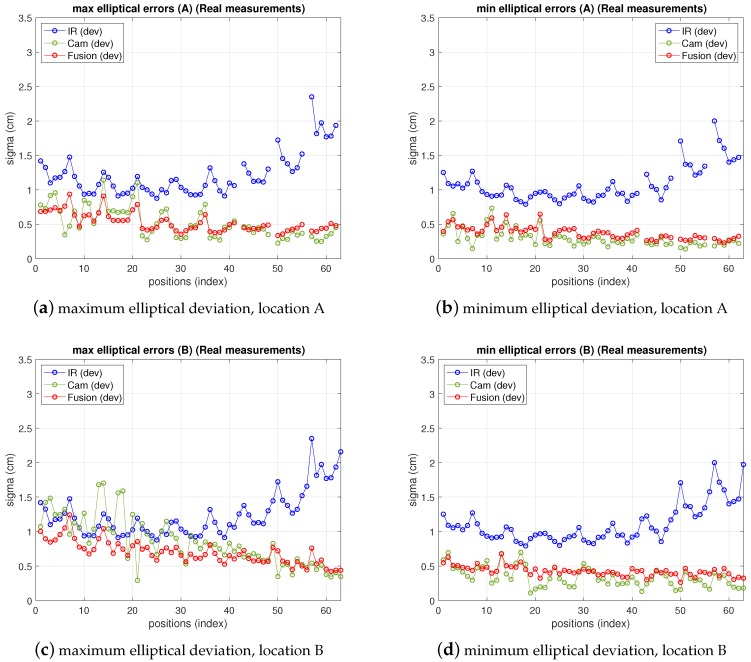
Elliptic deviations of IR, camera and fusion estimations from real measurements at locations A (top) and B (bottom). Left: maximum elliptical deviation; right: minimum elliptical deviation.

In Figure 11 the fusion errors in test-positions, obtained from the real measurements referred in the two previous points with the 5-anchor IR set and the camera in locations *A* and *B* are depicted, together with IR and camera ones. For easier figure reading only elliptical errors are displayed, as the information is more useful than x,y ones, because they provide the maximum deviations in the uncertainty ellipsoid axis (enough to define the dimensions of the 68% confidence elliptical area of the estimation). Thus, the elliptical standard deviations of camera, IR and fusion position estimate are shown as maximum and minimum deviations in a figure each. The abscissa axis of the figure represents the positions in the grid as stated in the setup in Figure 7. At every test position in the ground truth, the fusion estimation of such position is obtained as in (25), being the IR and camera position estimations in (25) obtained from the real measurements. The covariance matrixes in (25) are computed, also at every position, using the IR and camera models for variance propagation (from measurements to position estimate) as explained in their respective previous sections. These variances require the position coordinates to be computed. The position estimation obtained from the camera is used for this (the IR estimate, or the one with lowest variance, could be used too). Let us remember that, concerning the IR and camera variances, the former are obtained by means of an analytical expression derived from the IR model, while the latter are empirically inferred from the camera observations (afterwards the propagation to the covariance matrix of the camera position estimate is carried out by the analytical model). The choice of the pixelic deviation value to use in the fusion estimate must keep a balance between representing the real performance while being useful from the practical point of view. We follow a conservative criterion and choose the highest deviation values in Table 5: $\sigma_{x_{p}}=4.1$, $\sigma_{y_{p}}=4.5$, at *A*, and $\sigma_{x_{p}}=4.1$, $\sigma_{y_{p}}=3.9$ at *B*.

As can be seen, camera uncertainty (between some mm and 1.8 cm) is, in most positions, lower than IR one and the fusion deviations are much closer to the camera ones. The gaps in the traces in position A correspond to blind positions, as explained before.

**Figure 12:**
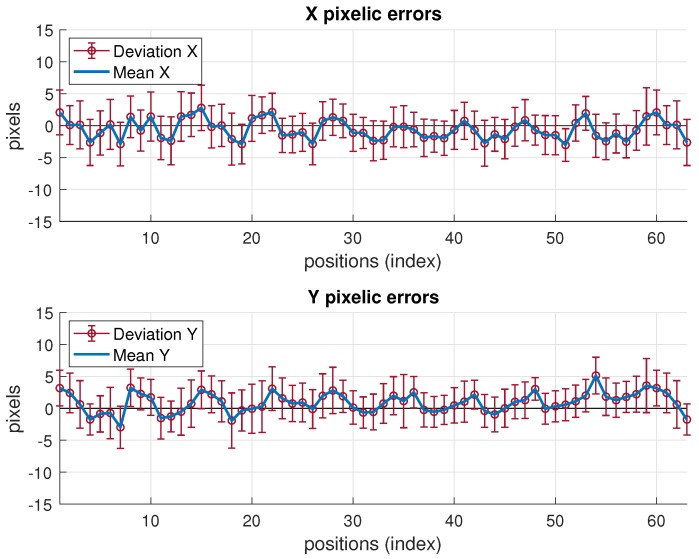
Pixelic errors in target detection at each position, all illumnation range.

Note that the fusion variance is slightly higher than the camera one at some points (namely, a little worse in the minimum elliptical direction, displayed in the image on the right). This is because the values of $\sigma_{x_{p}}$ and $\sigma_{y_{p}}$ chosen were the highest in all the grid, according to Table 5. Therefore, in many grid positions the variance is over dimensioned (as if working with a virtual distribution with worse variance in (25) at many positions). Proceeding in the opposite way, i.e., choosing the lowest values would lead to apparently better, but unrealistic, results as it would now overestimate the behavior at many points grids. The theoretical model predicts a fusion variance with lower values than the other two (IR and camera), but there is a differences between the model stated and the real behavior: in the model-based simulations two values of $\sigma_{x_{p}}$ and $\sigma_{y_{p}}$ were supposed to be the same for all the images, hence the propagation to the position met this condition. The real behavior shows different $\sigma_{x_{p}}$ and $\sigma_{y_{p}}$ at different positions. However, setting specific values at each position is not practical as, unlike the IR sensor, there is not a fully analytical model to express the variance as a function of target position. Selecting the value of the highest variance values along the whole grid is a good solution for the whole scene. Apart from this, the selection of $\sigma_{x_{p}}$ and $\sigma_{y_{p}}$ described before would match a theoretical model for unbiased Gaussian distribution of observations. However, the camera position estimates show a bias, as displayed in Figure 12, which is not taken into account by the previous approach. The values of the bias can be in the order of the deviations considered and, therefore, the total error assigned to camera observations is underestimated by the pure unbiased Gaussian assumption. Moreover, this bias is different at every grid-position (and camera location one too) with no systematic pattern, or at least it is not easy to model as it is highly dependent on environment parameters, geometrical configuration and image processing algorithm. Consequently, in order to take into account this contribution to error, now, the bias will be assimilated into the inferred $\sigma_{x_{p}}$ and $\sigma_{y_{p}}$ model parameters. Following again a conservative criterion, the highest values are determined from data represented in Figure 12. This deviates the model (AWGN assumption) from the real error performance (biased distribution) but this way the model is much more tractable. Aiming at having a simple procedure based on the model, two unique values $\sigma_{x_{p}}$ and $\sigma_{y_{p}}$ are selected from the information represented in Figure 12, for the whole grid and for all camera locations as well. As mentioned, this approach allows for having a useful model while matching the results satisfactorily, as shown next.

**Figure 13:**
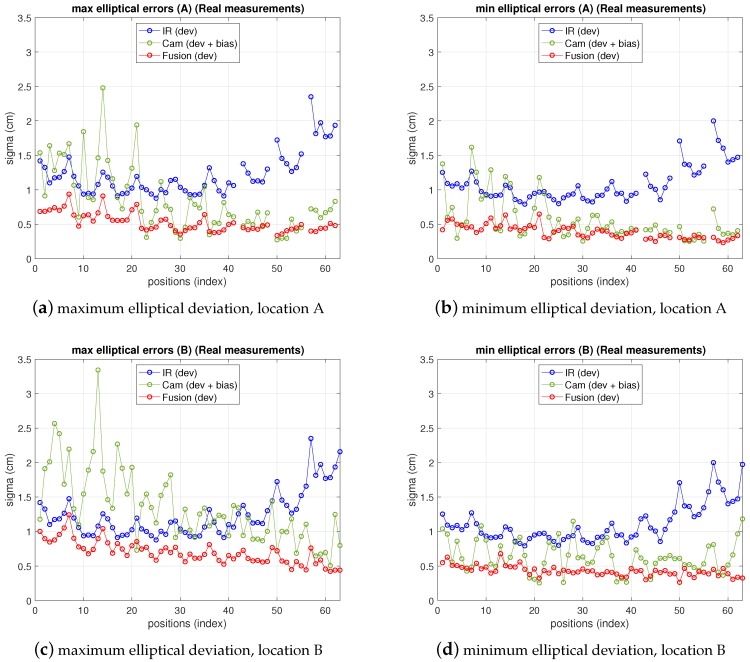
Errors for IR, camera (includes bias) and fusion estimations from real measurements at locations A (top) and B (bottom). Left: maximum elliptical deviation; right: minimum elliptical deviation.

**Figure 14:**
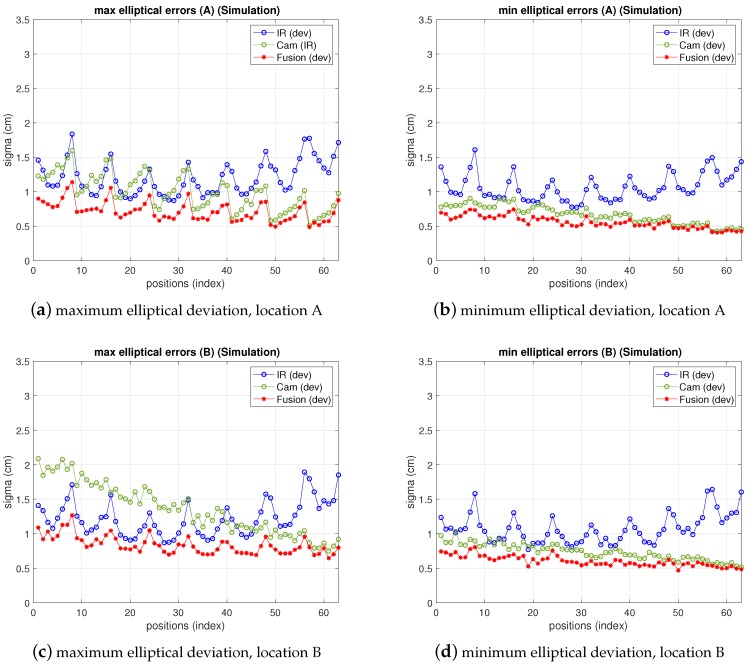
Simulation errors for IR, camera and fusion estimations, emulating locations A (top) and B (bottom). Left: maximum elliptical deviation; right: minimum elliptical deviation.

Figure 13 shows the results of IR, camera and fusion. The camera error has been redefined and displayed according to the discussion in the previous paragraph in a mixed manner, fitting better the real behavior: in the figure, the bias has been added to the deviation depicted in previous figures. On the other hand, the fusion variance, resulting from the choice of pixelic deviations as explained in the previous paragraph is below both IR and camera variances in most positions. Furthermore, comparing these results with simulation ones (shown in Figure 14), obtained as explained in Section 3, Section 4 and Section 5 with the model parameters set to the real values and $\sigma_{x_{p}}$, $\sigma_{y_{p}}$ chosen as explained, it can be seen that the (2σ) difference in the fusion (between measurements and model predictions) are within a difference of 0.4 cm considering the whole grid (see Table 6 and Table 7). The camera real behavior shows higher fluctuations than the model ones, while still remaining within a margin of 2.5 cm (mainly due to two points; on average keeps below 0.3 cm).

In summary, the model allows for having a simulation tool that facilitates testing the positioning space considered within a margin below 1 cm. Regarding precision achieved in target positioning, the whole system performs within a 95% (2σ) confidence error ellipse of 2.5 cm, enhanced with the strong robustness provided by fusion, compared with any of the two sensors working independently.

## 7. Conclusions

We have successfully developed an indoor positioning system with fusion of IR sensors and cameras (5 IR sensors and one camera). The achievable precision is in the cm level (maximum of 2.5 cm, 1.4 cm on average, within 95% confidence ellipse) in localization areas of 3×4 m, with IR sensors in a 3×3 m cell and a camera in possible locations up to a distance of 7 m from the target. It has been tested with measurements in a real setup, validating both the system performance and also the proposed models for variance propagation from IR and camera observations to the resulting fused position. Model and measurement fusion results agree within a margin of 0.4 cm (2.5 cm for camera, 1 cm for infrared), allowing for the models to be used as a valuable positioning systems simulation tool. Regarding positioning precision itself, while camera and IR ones (95% confidence margin), may rise up to 4.7 cm and 6.7 cm respectively, fusion improves precision reducing the deviations to 1.9 cm. On average, IR and camera present 2σ values of 2.5 cm and 2.7 cm; fusion lowers it to 1.4 cm. This means approximately a 48% precision improvement on average (in the maximum deviations it rises to about 72%). An average improvement of almost 50% may be quite convenient in some applications. Moreover, as important as precision performance, the sensor fusion strategy provides the system with high robustness as, in case one sensor fails or its performance worsens, the fused position precision gets closer to the other one. The system also works under changing illumination. The camera performs satisfactorily with very low illumination levels and also with floor brightness (high artificial illumination conditions). Furthermore, IR and camera provide complementary behavior with respect to light conditions, as in dark conditions the camera might not see the scene but the IR system would perform correctly, while under high illumination levels the IR receivers could saturate but the camera would still capture the landmarks. The last two features pointed out add robustness and reliability to the system. The proposal shows promising perspectives in terms of scalability to large real spaces because the system developed offers wide coverage with a small number of low cost devices (less than five IR anchors is also valid) and high precision features, with the camera enhancing the decrease of IR precision beyond 5 m. Locating areas are expected to be notably enlarged without increasing the number of devices, although further tests are necessary for proper assessment. This could be attained by placing the camera at longer distances and by improvement of the IR precision (increasing sensitive area, frequency and emitted power is feasible). From the low level point of view, this will imply a challenging design effort to balance the distance between devices so as to keep cost as low as possible and still have acceptable coverage and precision (increasing the distance will reduce SNR). Careful selection of new devices with higher working frequency (which also enhances precision) and wider field of view, without engaging the response time, is a key aspect in this low level tradeoff. With respect to response time requirements (under real time navigation conditions), a more restrictive signal filtering (IR) and resolution increase (camera) would increase SNR (precision) but would make the system slower. Considering all these aspects, the final system should also be tackled together with the aid of odometry (which can also be tackled with a fusion approach). Finally, we have proposed a BLC with five receivers but the minimum localization unit needs three, although precision worsens. If the precision requirements relax the number of receivers could be lower (not necessarily the fusion precision, unless the camera worsens too).

From the theoretical point of view, the IR model for variance propagation is completely analytical and matches real results very closely at every target position in unbiased scenarios, i.e., mainly multipath free (or MP canceled). The camera one is semi-empirical, the variance of observations being deduced from the measurements. A completely analytical model for the camera is not easy to define as some stages are not accessible as to link variance with position (mainly the detection algorithm). Nevertheless, unlike the IR system, the determination of the pixelic variances, in order to obtain the H matrix of the model, is very easily achieved with one single picture-burst of the whole scene (with IR, this would need a large amount of measurements, successively at every position in the grid). Moreover, although this task has been carried out offline, under real navigation performance, it could be easily carried out online. In summary, a good tradeoff solution has been found for determining the variance of the camera observations, which represents within 1 cm the real behavior in the whole grid. The camera bias is very position-dependent and not easy to model, but it has also been successfully integrated in the procedure. Regarding IR, as said, AWGN assumption requires multipath free scenario what requires cancelation techniques. Otherwise, multipath errors can grow up to the m level in unfriendly (though common) scenarios.

A next challenge is improving the model for the camera, by going further in the analytical link between variance and target position. In addition, it will be important to tackle the problem from an estimator-based approach, addressing the approximations assumed here with respect to the position-depending variances in the ML solution, and investigating the camera bias and IR multipath contribution to integrate them in the models. Finally, exploring sensor cooperation capabilities together with data fusion will be interesting when applied to real applications.

## Figures and Tables

**Table 1:** Indoor positioning review. Cost: Low (L), Low-Medium (L-M), Medium (M), Medium-High (M-H) and High (H).

Ref.	Application	Acc.	Technology	Cost	Pros.	Cons.
Jun [7]	Autonomous robots	1 cm	US TOF + RF link for sync	M	Accurate	Small (validated) coverage
Jung [33]	Not mentioned	1 cm	Optical, TDOA	M	Accurate	Only simulation
Raharijaona [11]	Autonomous robots	2 cm	Optical, AOA, CDMA	M	Accurate	Needs dense lighting infrastructure
Wang [34]	H-speed indoor communications	2.5 cm	Optical, AOA + RSS	M	More accurate than standard VLC based	Ad-hoc receiver
Zhang [15]	Not mentioned	5 cm	Optical, RSS	L	Accurate	Only simulation
Lee [36]	Augmented reality	1–5 cm	Vision, ad-hoc IR reflecting landmarks	L-M	Independent of illumination	Poor validation
Sani [24]	Nav. and landing drone	6 cm	Cooperation Cam + IMU. ArUco markers + PnP, IMU + Kalman Filter	L	Works without cam	Controlled from ground station
Zhu [43]	Not specified	<10 cm	Pseudolites. GNSS-like ranging (code/phase tracking) + complex correction algorithm	M-H	Accurate	Only simulation results
Yun-Ting [32]	Autonomous robots	<10 cm	2D laser scanning + feature matching with map	M	No infrastructure needed	Requires previous acquisition and classification of map features
Kumar [35]	UAVs	<10 cm	2 × 2D laser scanners + IMU (for heading estimation). SLAM	M	No infrastructure needed	Cost
Paredes [14]	UAV	<10 cm	US TOF (CDMA) + TOF camera for initialization	M-H	Accurate, fast	Expensive
Kuo [39]	LBS	10 cm	Vision + optical (AOA from leds on camera, ID from leds with CDMA)	L	Position + orientation. Low cost, good validation, smart use of camera for demodulating	Not clear how dense the infrastructure should be
Nakazawa [38]	LBS, human navigation	10 cm	Vision + optical (AOA from leds on camera, ID from leds with CDMA)	M	Good trade-off accuracy VS range	Ad-hoc receiver
Garcia [12]	Loc. in complex environments	10 cm	UWB TOF + SW multipath mitigation	M	Good scalability	Nodes are expensive
Tiemann [13]	UAVs	10 cm	UWB TOF + SW multipath mitigation	M	Good scalability	Nodes are expensive
Montero [22]	Robots localization	5–13 cm	Phone cam, Landmark + Fern descriptor	L	Non invasive	Synthetics data
Alatise [29]	Autonomous robots	5–14 cm	Cam(SURF + RANSAC) + IMU. Fusion(EKF)	M	Accurate	Field of view is limited
Pizarro [16]	Autonomous robots	<20 cm	Reconstruction (structure-from-motion) + odometry	M	Non-supervised method	No multiple robots tested
Xin [44]	Not specified	<20 cm	Pseudolites. GNSS-like ranging (code/phase tracking) + ambiguity resolution of carrier phase for enhanced accuracy	M-H	Good trade-off accuracy vs range	Requires independent initialization
Xu [27]	Autonomous robots	<25 cm	Cooperation. Edges of regular ceiling + Hough + LMS + RANSAC + Odometry	M	Non invasive	Cumulative errors
Losada [25]	Autonomous robots	<30 cm	Multi-camera sensor. Background model + Generalized Principal Components Analysis	M-H	Localization of multiple mobile robots	No real time performance
Lee [37]	Autonomous robots	<35 cm	Vision (natural landmarks) + IR ranging	L	Robustness	Only simulation
Xu [20]	Autonomous robots	<40 cm	Cooperation. Cam + CNN (coarse loc.), LIDAR (fine loc.)	H	Recovery from localization failures	Pre-trained Network
Duraisamy [30]	Autonomous driving	<0.6 m	Fusion stereo cam + Radar + Lidar. Weighted sum of the covariances	H	Tested in real traffic condition	Fusion accuracy dependent on the sensor inputs
Luo [6]	Robot/human localization	0.6 m	Fusion multi WiFi PIR + Cramér–Rao Bound + triangulation(RSSI)	L	Integrated wireless and PIR sensor (WPIR)	Requires at least three sensor nodes
Chen [21]	Robot/human localization	0.25–1 m	RGB-D cam + CNN (coarse loc.), ORB-Features (fine loc.)	M	Indoor and outdoor	Needs geotagged images
Guan [3]	Human localization	<1 m	Phone cam, Landmark + SURF (offline.), SURF + Match + Homography (online)	L	Reduces latency	Needs offline image database
Mohebbi [31]	Loc. for multiple occupants	1.8 m	Motion sensors + BLE beacon, fusion using a weighted sum	L	Recognizes activities	Low accuracy

**Table 2:** Setup characteristics.

IR System		Camera		BLC and Test Conditions	
Emitter	IRED: SFH 4231 (OSRAM)	Sensor	IMX 219 PQ CMOS (Sony)	Dimensions	4×3 m
Emitted Power (Pe)	100 mW/sr	Resolution	3280×2464	Test-Grid ($X_{1}$ to $X_{63}$)	9×7 target positions in 50 cm grid steps
Emitted signal	IMDD IR signal modulated at 4 MHz	Transfer rate	10 fps	IR-anchors	5 anchors in 3×3 IR deployment (4 at corners, reference in the center)
Detector	Photodiode: PIN100-11-31-221 (API)	Lens	$1/4^{\prime \prime }$, 24×25×9 mm	Camera Locations	4 locations ($CL_{1}$ to $CL_{4}$)
Sensitive Area (As)	5 mm${}^{2}$	HW	Raspberry Pi 3 Model B	H-markers positions	16 ArUco markers
Responsivity (R)	0.65 A/W	Algorithm	Corner detection (Shi-Tomasi) plus centroid search	Number of observations per position	200
i-v conversion gain ($G_{A}$)	$33\times 10^{3}$ V/A	Landmark $X_{i}$	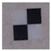	illumination conditions for camera	4 illumination levels
BP filter gain ($K_{F}$) and I/Q gain ($K_{I/Q}$)	1 (V/V)	13×13 cm H-markers (ArUco)	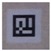		
Noise Power	$6\times 10^{-14}$ V${}^{2}$/Hz				
Noise eq. BW	30×π/2 Hz				

**Table 3:** Infrared. Estimation-clouds shape and measurements precision.

Shape Indicators			Dispersion Indicators (95% Confidence Ellipsoid)			
${\mathrm{DI}}_{x}$	${\mathrm{DI}}_{y}$	$\bar{\mathrm{CI}}$	$\max_{\mathrm{BLC}}\left(2\sigma_{{\mathrm{ellip}}_{\max}}\right)$	${\bar{2\sigma }}_{{\mathrm{ellip}}_{\max}}$	$\max_{\mathrm{BLC}}\left(2\sigma_{{\mathrm{ellip}}_{\min}}\right)$	${\bar{2\sigma }}_{{\mathrm{ellip}}_{\min}}$
7.2%	5.7%	86.2%	3.5 cm	2.8 cm	3.4 cm	2.5 cm

**Table 4:** Camera. Estimation-clouds shape and measurements precision.

	Shape Indicators			Dispersion Indicators (95% Confidence Ellipsoid)			
**Cam. Locations**	${\mathrm{DI}}_{x}$	${\mathrm{DI}}_{y}$	$\bar{\mathrm{CI}}$	$\max_{\mathrm{BLC}}\left(2\sigma_{{\mathrm{ellip}}_{\max}}\right)$	${\bar{2\sigma }}_{{\mathrm{ellip}}_{\max}}$	$\max_{\mathrm{BLC}}\left(2\sigma_{{\mathrm{ellip}}_{\min}}\right)$	${\bar{2\sigma }}_{{\mathrm{ellip}}_{\min}}$
**1**	47.5%	30.6%	50.8%	1.3 cm	0.7 cm	0.7 cm	0.5 cm
**2 (A)**	38.6%	26.8%	60.1%	2.3 cm	0.9 cm	1.5 cm	0.7 cm
**3**	49.9%	18.9%	45.3%	2.7 cm	1.4 cm	1.0 cm	0.8 cm
**4 (B)**	31.8%	7.1%	43.9%	3.4 cm	1.6 cm	1.4 cm	0.9 cm

**Table 5:** Pixelic sigmas.

Cam. Locations	$\min(\sigma_{x_{p}})$	$\bar{\sigma_{x_{p}}}$	$\max(\sigma_{x_{p}})$	$\min(\sigma_{y_{p}})$	$\bar{\sigma_{y_{p}}}$	$\max(\sigma_{y_{p}})$
**1**	1.859	3.013	4.023	1.854	2.375	4.788
**2 (A)**	0.738	3.021	4.066	1.472	3.104	4.491
**3**	1.055	2.628	3.636	1.162	2.004	5.560
**4 (B)**	0.625	2.550	4.146	1.272	2.573	3.870

**Table 6:** Summary of precision for IR, camera and fusion (real measurements).

	IR		Camera		Fusion	
**Cam. Locations**	$\max_{\mathrm{BLC}}\left(2\sigma_{{\mathrm{ellip}}_{\max}}\right)$	${\bar{2\sigma }}_{{\mathrm{ellip}}_{\max}}$	$\max_{\mathrm{BLC}}\left(2\sigma_{{\mathrm{ellip}}_{\max}}\right)$	${\bar{2\sigma }}_{{\mathrm{ellip}}_{\max}}$	$\max_{\mathrm{BLC}}\left(2\sigma_{{\mathrm{ellip}}_{\max}}\right)$	${\bar{2\sigma }}_{{\mathrm{ellip}}_{\max}}$
**2 (A)**	4.7 cm	2.4 cm	4.9 cm	1.7 cm	1.9 cm	1.0 cm
**4 (B)**	4.7 cm	2.4 cm	6.7 cm	2.7 cm	2.5 cm	1.4 cm

**Table 7:** Summary of precision for IR, camera and fusion (simulation).

	IR		Camera		Fusion	
**Cam. Locations**	$\max_{\mathrm{BLC}}\left(2\sigma_{{\mathrm{ellip}}_{\max}}\right)$	${\bar{2\sigma }}_{{\mathrm{ellip}}_{\max}}$	$\max_{\mathrm{BLC}}\left(2\sigma_{{\mathrm{ellip}}_{\max}}\right)$	${\bar{2\sigma }}_{{\mathrm{ellip}}_{\max}}$	$\max_{\mathrm{BLC}}\left(2\sigma_{{\mathrm{ellip}}_{\max}}\right)$	${\bar{2\sigma }}_{{\mathrm{ellip}}_{\max}}$
**2 (A)**	3.7 cm	2.7 cm	3.2 cm	1.8 cm	2.3 cm	1.4 cm
**4 (B)**	3.7 cm	2.7 cm	4.2 cm	2.4 cm	2.5 cm	1.6 cm
